# Prevalence of Bacterial Vaginosis and Its Association with Risk Factors among Nonpregnant Women: A Hospital Based Study

**DOI:** 10.1155/2018/8349601

**Published:** 2018-03-05

**Authors:** Eliza Ranjit, Bijendra Raj Raghubanshi, Smrity Maskey, Pramila Parajuli

**Affiliations:** ^1^Department of Microbiology, St. Xavier's College, Maitighar, Kathmandu, Nepal; ^2^Department of Microbiology, KISTMCTH, Lalitpur, Kathmandu, Nepal; ^3^Department of Gynaecology/Obstetrics, KISTMCTH, Lalitpur, Kathmandu, Nepal

## Abstract

Bacterial vaginosis (BV) is an ecological imbalance of the vaginal microbiota affecting mostly women of reproductive age group. This study was carried out among 160 nonpregnant women registered at the Outpatient Department of Gynaecology/Obstetrics of KIST Medical College Teaching Hospital, Imadol, Lalitpur, Nepal, from November 2014 to May 2015. The aim of the study was to assess the association of the risk factors with BV and analyze the type of bacteria associated with BV. Nugent's scoring method was used for diagnosis of BV in this study. The overall prevalence of BV was 24.4% among symptomatic patients. Douching was statistically related to BV (*P* = 0.015). Also, BV was significantly associated with consistency (*P* = 0.0001), odor (*P* = 0.02), and amount of abnormal vaginal discharge (*P* = 0.09). Contraceptives users on anatomical sites were found more prone to BV than those who did not use contraceptives on anatomical sites.* Pseudomonas *spp.,* Escherichia coli, Acinetobacter *spp.*, Proteus *spp.*, Klebsiella *spp.*, Neisseria gonorrhoeae, Enterobacter *spp.*, Citrobacter *spp.*, Staphylococcus aureus, *Coagulase-Negative Staphylococci (CoNS), and* Streptococcus agalactiae *were associated with BV and out of those* Lactobacillus *spp. was the predominant organism. The higher prevalence of BV among symptomatic patients indicates interventions should be applied to reduce the incidence of stillbirth, abortion, and sterility.

## 1. Background

Vaginitis is an inflammation of the vagina, usually characterized by any of the following: vaginal discharge containing many white blood cells (WBCs), vulvar itching, vulvar irritation, vaginal odor, vaginal erythema, dyspareunia, and dysuria [1, 2]. The three most common causes of vulvovaginitis are bacterial vaginosis (BV), being the most prevalent one, followed by candidiasis and trichomoniasis [3]. BV is a common vaginal infection that occurs mostly in women of child-bearing age [4].

BV is a clinical condition characterized by a thin, gray/off-white, homogenous, malodorous adherent vaginal discharge which is more noticeable after intercourse and menses, having pH > 4.5. Fishy odor is noticed on addition of 10% potassium hydroxide to the vaginal fluid (whiff test), and the presence of clue cells, a few or no lactobacilli, and small number (<1/hpf) of polymorphonuclear leucocytes (PMNs) are also the characteristic features of BV [5].

Many cases of BV remain asymptomatic or present with only malodorous vaginal discharge with no inflammatory complaints [6]; thus BV is therefore referred to as “vaginosis” and not “vaginitis” [7].

Lactic acid produced by the normal flora,* Lactobacillus *through hydrogen peroxide (H_2_O_2_) production, is attributed to the acidic milieu of the vagina. This provides a local defense mechanism by inhibiting the growth of other organisms. Change in the normal vaginal flora causes change in pH simultaneously, which allows a variety of anaerobes and facultative bacteria to overgrow and cause chronic infection as well as abnormal vaginal discharge [5, 8]. Lactobacilli also produce antimicrobial substances like lactic acid, H_2_O_2_, and bacteriocin to promote a healthy ecosystem in the vagina thereby suppressing the growth of pathogens [8, 9].

Besides* Lactobacillus *spp., other bacteria are also frequently found in the vaginal microbiota of healthy women. The organism found are Gram positive cocci and Gram negative rods, namely,* Streptococcus *spp.,* Staphylococcus *spp., and members of Enterobacteriaceae, mostly* E. coli *in addition to other anaerobic species e.g., Bacteroides spp.,* Bifidobacterium *spp.,* Propionibacterium*,* Fusobacterium *spp.,* Peptococcus *spp.,* Prevotella *spp.,* Veillonella *spp.,* Peptostreptococcus *spp.,* Atopobium vaginae*,* Gardnerella vaginalis*,* Ureaplasma urealiticum*,* Mycoplasma hominis, *and* Mobiluncus *[10, 11]. When the population level of lactobacilli decreases below a critical level, these opportunistic bacteria may overgrow becoming the dominant species in the environment [12].

In pregnancy, vaginal infections can be associated with drastic complications both to the mother and the neonate [13], leading to gynaecologic and obstetric complications [12]. BV also increases the risk of acquiring Human Immunodeficiency Virus (HIV) and Sexually Transmitted Diseases (STDs). BV may be considered as Sexually Enhanced Disease (SED) rather than STDs, in which the frequency of intercourse plays a key role [14]. Hence, emphasis to explore primary preventive strategies needs to be given more priority.

Preventive strategies target the risk factors or behaviors for a disease. Studies suggest that BV is associated with a number of risk factors and behaviors, including age, marital status, employment status, occupation, recent antibiotic use, decreased estrogen production of the host, douching, sexual activity, lower age of first intercourse, more frequent episodes of receptive oral sex, spermicide use, STDs, working as a sex worker, smoking, alcohol intake, stress, contraceptives used, frequency of vaginal intercourse, and race/ethnicity [15, 16]. A number of observational studies have reported that women using hormonal contraceptives have a reduced risk of recurrence to BV [17].

In particular, among the most prominent risk factors, sexual behavior and vaginal douching are modifiable risk factors [14]. However, few studies have determined the prevalence of BV and associated risk factors among women in Nepal. Therefore, this study was undertaken to assess the association of the risk factors with BV and analyze the type of bacteria associated with BV.

## 2. Methods

### 2.1. Study Design

A descriptive cross-sectional study was conducted among 160 nonpregnant women, attending KIST Medical College Teaching Hospital over a period of six months from November 2014 to May 2015.

### 2.2. Ethical Approval

Ethical statement was approved by the ethical committee of KIST hospital and a verbal consent of each of the patient was obtained. Questionnaires were used to collect sociodemographic, behavioral characteristics and medical, reproductive, and sexual history information.

### 2.3. Sample Collection and Processing

A nonlubricated speculum was inserted into the vagina to collect discharge and a physical examination of the discharge was performed to observe its form, color, consistency, and odor. Then, two vaginal samples were taken with sterile cotton-tipped swabs from the lateral and posterior vaginal fornix.

**Figure 1:**
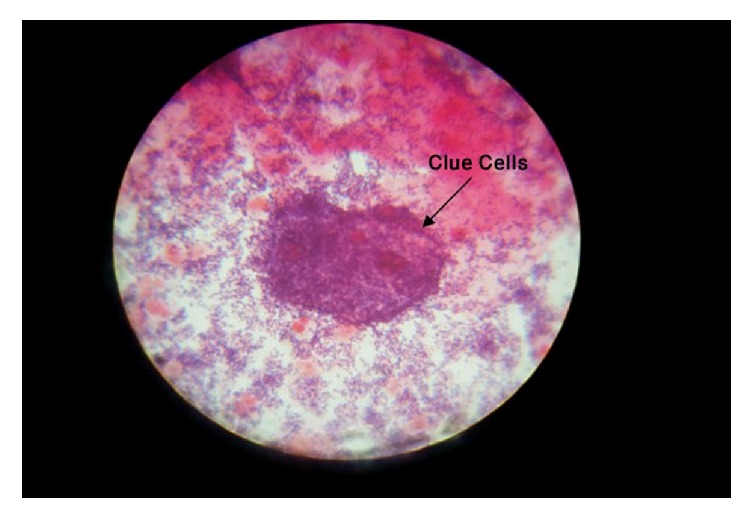
Clue cells on Gram-stained smear.

**Figure 2:**
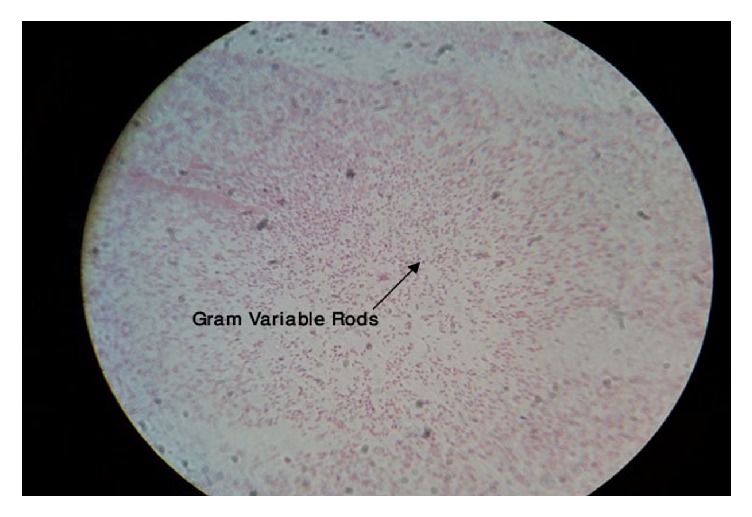
Gram-stained vaginal smears with Gram variable rods.

**Figure 3:**
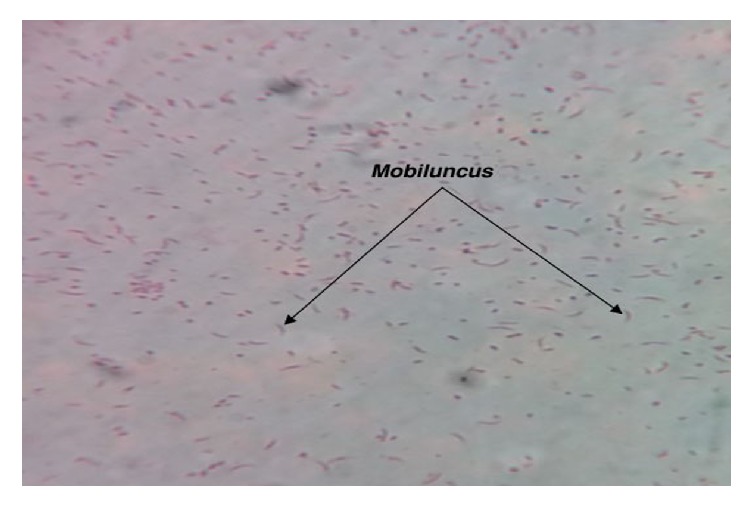
Direct Gram stain of high vaginal swab showing Gram negative curved rods-*Mobiluncus.*

One of the swabs was transported in sterile capped test tube to the Microbiology Laboratory for aerobic and anaerobic cultures in MacConkey agar, Blood agar, and Chocolate agar for identification of bacterial isolates. The former two agars were inoculated aerobically at 37°C for 24 hours and later anaerobically at 37°C in candle jar for 24 hours. Another swab was used for making a smear for Gram staining (Figures 1, 2, and 3) and direct wet mount microscopy. The Gram-stained slides were examined under oil immersion objective (1000x magnification). Then, the etiological agent and normal flora on the Gram-stained smear were counted and were scored according to the standardized Nugent's scoring method [18]. (Table 1).

The wet mount was performed to view the presence of motile oval flagellated protozoa,* Trichomonas vaginalis*, clue cells, and white blood cells. For yeasts, Gram staining and germ tube test was done.* Candida albicans *was identified by its ability to produce germ-tubes when incubated in serum for 2 hours at 37°C.

### 2.4. Statistical Analyses

A descriptive analysis was performed to calculate prevalence of BV and microorganisms. Chi square values were calculated at 5%  (*α* = 0.05) level of significance using the Statistical Package for Social Sciences (SPSS-16).

## 3. Results

Upon examination of 160 nonpregnant women with symptomatic vaginal discharge, the overall prevalence of BV was 24.4% based on Nugent's scoring system.

The highest number of BV cases was seen among 30–40 years' age group (8.8%) and least BV cases were seen in patients with age group of 10–20 and 50–60 years (1.3%). Unmarried women were more prone to BV, that is, (100%), followed by married women (24.2%). Illiterate women were highest with 21.9% of total respondents. The prevalence of BV was high among farmers (38.9%) and low in those who were involved in business (14.7%). Higher rate of BV was seen among other ethnicity (57.1%) followed by Dalit (50%), Brahmin (25%), Janajati (22.7%), and Chhetri (19.4%) among all respondents. Hence, women of age group 30–40 years, unmarried, illiterate, farmers, and belonging to other ethnic groups were more prone to BV (Table 2). However, the result was not statistically significant at *α* = 0.05.

The prevalence of BV was found higher in daily smokers (30%) while low rate was reported among occasional smokers (0%). Daily alcohol users had the highest prevalence of BV of 38.5% while the least prevalence of 24% was recorded among those who never drank alcohol and 16.7% were occasional alcohol users. BV was seen very high among nonvegetarians with 25.2% whereas it was only 15.4% among vegetarians. The risk of BV was higher among those who used condom daily (42.9%) than those who used condoms sometimes (29.2%). Women having daily douching habit were more likely to have BV (32.1%) than in women with occasional douching habit (23.7%) and the difference was significant (*P* = 0.015) (Table 3).

The highest prevalence of BV was found among women whose partner had undergone tubectomy (50%) and least prevalence was found among OC pills users (9.1%) (Table 4).

In terms of consistency of abnormal vaginal discharge among BV cases, occurrence of the thin discharge had the highest prevalence (39.8%) while the least was among women with normal discharge (4.9%). Foul discharge was reported in (29.9%) of patients while nonfoul discharge was found in (14.3%) of patients. Excess discharge was observed in patients with BV (30%) while scanty discharge was observed in (18.7%). Women with BV were found to be associated with consistency, odor, and amount of vaginal discharge (*P* < 0.05) (Table 5).

A total of 99 bacteria belonging to 14 different species were isolated from vaginal samples, which are tabulated in Table 6. Among the isolates, 41 (32%) were Gram negative and 58 (45.3%) were Gram positive. Other isolates including yeasts and parasites were 29 (22.7%). Of the Gram negative bacteria,* Pseudomonas *spp.,* E.coli, *and* Acinetobacter *spp. were predominant.* Lactobacillus *spp. were the dominant Gram positive bacteria found in vaginal samples.

## 4. Discussion

Globally, BV is a common genital problem among women seeking gynaecological care. The prevalence rate of BV was found to be 24.4% by Nugent's method. Modak et al. [19] in India reported a similar result, providing a prevalence rate of 24%. Higher prevalence rates of BV than those in the present study were also reported by Gad et al. [20] in Egypt (33%). The variation in the findings might be due to population size, methods of analysis, geographic distribution, and socioeconomic and behavioral differences in the studied population.

The present study revealed that the prevalence of BV was high among women of age group 30–40 years (8.8%) and least for 10–20 and 50–60 years' age groups (1.3%). However, the difference between them was not statistically significant. Bhattarai [6] in Nepal observed the highest prevalence of BV among the age group of 31–40 years (60.16%) and least among those below 20 years of age and 51–60 age group (33.33%) which was similar to the above study. Also, Garba et al. [21] in Nigeria found BV to be most prevalent among 26–30 age group (35.8%) and least in >40 age (10.5%). The highest prevalence in the age group 30–40 years might be due to the age being the most reproductively active age group and high sexual exposure at this age.

According to this study, unmarried women were at higher risk (100% tested positive) compared to married women (24.2%). The finding of the study is contradicted by Gad et al. [20] in Egypt. However, several studies have documented the occurrence of BV in sexually inactive females or virgins [22, 23]. This provides evidence that sexual activity is not a prerequisite for BV. The change in lifestyle, improper perineal care, food habits, tight clothing, lack of attention towards menstrual hygiene, and sedentary factor might be the reasons for the acquisition of BV in unmarried women.

Similarly, illiterate women had the highest BV prevalence of 29.1% which differs from Ibrahim et al. [24] who recorded the highest prevalence of 54% in those with primary education in Nigeria. The low economic status, lack of education, lack of a female consultant at the health service center, hesitance to approach medical service, and sociocultural structure might be the cause of higher prevalence of BV among less educated women.

In the study, higher rate of BV was seen among farmers which is similar to the finding of a study undertaken by Garba et al. [21] in Nigeria who reported the highest prevalent BV (52.6%) in farmers. Again, the magnitude of BV was higher among women of other ethnicity, which is different from a survey conducted in Nepal by Manandhar et al. [25] that reported the highest prevalence of BV in Dalits. A combination of environmental, contextual and institutional factors, and chronic stress may contribute to this disparity [15].

Moreover, the study revealed that daily smokers were more prone to BV than those who never smoke but the difference between them was not significant (*P* > 0.05) which is contrary to the results of a study carried out by Manandhar et al. [25] in Nepal. Risk of BV increases as the number of cigarettes smoked per day increases. Various chemical constituents of cigarette smoke alter the vaginal microflora or may act by depleting Langerhans cells in cervical epithelium leading to local immunosuppression, thus causing BV [26]. The study showed daily alcohol users had a higher rate of BV (38.5%) than those with an occasional consumption of alcohol but this association was not significant. However, the finding is consistent with the report of Hellberg et al. [27] in Sweden which stated that alcohol use was not significantly associated with BV. Smoking cigarettes and alcohol intake causes depletion of hydrogen peroxide–producing lactobacilli, therefore increasing the risk of BV. This study showed nonvegetarians had high BV occurrence (25.2%) than vegetarians (15.7%) which was statistically insignificant (*P* > 0.05). The finding is similar to the finding by Manandhar et al. [25] in Nepal. High fat intake, particularly saturated fat, may increase vaginal pH, thereby increasing the risk of BV [28].

In this study, the prevalence of BV was higher among women whose partners were daily condom users than those who were not. However, no significant association was found. The findings are more consistent with the study conducted by Mascarenhas et al. [4] in Brazil who reported no association between BV and condom use. One possible explanation for this is that condoms may cause irritation giving the bacteria a way to get into the vagina and increase risk of having BV.

It was observed that 32.1% of the BV positive cases were among women having a daily vaginal douching habit and 23.7% were found positive on those women having the habit of douching sometimes. This confirms that vaginal douches represent a risk factor for BV acquisition. A previous study also suggested a strong association between vaginal douching and BV [3, 19]. The lack of effort and awareness on the health hazards of this incorrect practice might be regarded as the cause of higher possibilities of BV among women having vaginal douches habit.

The study also clarifies that the chance of getting BV increases in those women who have used contraceptives on anatomical sites than those who had not. So, use of contraceptives on anatomical sites may cause imbalance in vagina flora and leads to BV. Lowest rate of BV was seen among women using OC pills (9.1%). The estrogen increases the glycogen content of vaginal epithelial cell activity, in turn inhibiting the in vitro growth of certain bacteria, which may result in low risk for BV [16]. Similarly, the study also agrees with Thulkar et al. [29] in India who reported protective effects of hormonal contraceptives against BV.

In this investigation, consistency, odor, and amount of vaginal discharge were found to be statistically significant with BV (*P* < 0.05). This finding corresponds with previous study conducted by Garba et al. [21] in Nigeria. White colored vaginal discharge had the highest prevalence than gray colored discharge and was found not to be associated with diagnosis of BV. There is no specific color of vaginal discharges for the diagnosis of BV; experts varied in opinion but it is important to know that women with BV present abnormal vaginal discharges and the vaginal discharges differ in the consistency, Garba et al. [21]. Abdominal pain and itching symptoms were found in 26.6% and 23.2%, respectively, which was consistent with the result reported by Nzomo et al. [30] in Kenya. This study registered no significant relationship between abdominal pain, itching, and BV.

Of the 128 isolates, 35 isolates belonged to* Lactobacillus *spp. and 93 isolates were of opportunistic pathogens.* Lactobacillus *spp. was isolated as the most predominant species and accounted for 27.3% of the total bacterial isolates. Higher prevalence of* Lactobacillus *in this study also resembles the study done by Razzak et al. [31] in Iraq and Larsen and Monif [32] in Omaha. Therefore,* Lactobacillus *plays a protective and probiotic role in treating and preventing vaginal infection by producing antagonizing compounds and is regarded as safe for human [31].

The second common pathogen was* Pseudomonas *spp., accounting for 7.8% BV cases, followed by many other Gram negative bacteria, namely,* E. coli, Acinetobacter *spp.*, Proteus *spp.*, Klebsiella *spp.*, N. gonorrhoeae, C. koseri, and Enterobacter *spp. But, the findings of the study contradict with the studies undertaken by Razzak et al. [31] in Iraq, Marrazzo et al. [33] in Seattle, and Larsen and Monif [32] in Omaha.* Pseudomonas *spp. is potentially opportunistic bacteria within the vagina and may become increasingly prevalent upon minor alterations of the vaginal environment. It is considered as primary pathogen in compromised hosts, hospitalized patients, and complicated urinary tract infection [34]. Collectively, Mumtaz et al. [35] suggest that the presence of members of faecal flora in the vagina is attributed to unhygienic bowel practices.

The most common Gram positive cocci were* Staphylococcus aureus *and* Streptococcus agalactiae *in the study. The incidence was found to be 5.5% of total bacterial isolates each. This is consistent with the findings of Maghsoudi et al. [36] in Pakistan, Tiyyagura et al. [37] in India, and Al-Mousawi et al. [38] in Iraq. The vaginal mucosa colonized by* S. aureus *predisposes them to Toxic Shock Syndrome (TSS). The second most prevalent organism among Gram positive cocci were CoNS and* Enterococcus *spp. accounting for 3.1% and 3.9%, respectively. The finding is similar to the study conducted by Masood et al. [39] in Pakistan. CoNS is also a common vaginal colonizer. It continues to be the most important bacterial cause of bacterial sepsis and meningitis in newborns. Enterococci are an opportunistic pathogen and can cause infection when the immune system is impaired.

Regarding the frequency of yeasts and parasites isolated from patients,* Candida albicans *(2.3%) and other* Candida *spp. (9.4%) were reported. The finding is inconsistent with the reported prevalence of candidal infection by Al-Muk and Hasony [40] in Iraq and similar to Maghsoudi et al. [36].* T. vaginalis *was observed in 14 cases (10.9%) by wet mount preparation. These are nearly similar to the study reported by Begum et al. [5] in Dhaka (8%).

The levels of these various organisms vary in each woman [41]. The results showed that the presence of lactobacilli together with other opportunistic pathogens may be due to several factors like effects of antibiotics, type of incubation (as some* Lactobacillus *spp. are unable to produce some defense factors under anaerobic incubation), and antagonism among lactobacilli species to maintain dominance [31]. Also, some studies have reported that there can be chance of overlap of BV and aerobic vaginitis, leading to a mixed condition, but whether one condition can evolve into the other has not yet been determined [42].

## 5. Conclusion

The study suggests that using contraceptives on anatomical sites may confer a higher risk for BV. Some factors, especially vaginal douching, may increase the risk of BV.* Lactobacillus *spp. were the predominant isolates found in the vaginal sample followed by a number of Enterobacteriaceae members and Gram positive bacteria. This finding suggests that the colonization of facultative anaerobes is also more likely a consequence in vaginal ecology. Limited studies on BV have been performed in Nepal. So, similar studies must be carried out in order to improve the health status of women, thereby preventing the risk posed towards BV.

## Figures and Tables

**(a):** 

Lactobacilli	Score	*Gardnerella*, Bacteroides	Score	Curved gram negative bacilli	Score	Sum = ^*∗*^N-score
30 or >	0	0	0	0	0	0
5–30	1	<1	1	<1	1	3
1–4	2	1–4	2	1–4	2	5
<1	3	5–30	3	5–30	3	8
0	4	30 or >	4	30 or >	4	10

**(b):** 

^*∗*^Interpretation of Nugent Score		
If N score is	And	Then report
0–3		Normal
4–6	Clue cells not present	Intermediate
4–6	Clue cells are present	Bacterial vaginosis
>7		

**Table 2:** Sociodemographic characteristics of sample distribution.

	Bacterial vaginosis			*P* value
	Number (160)	Positive number (%)	Negative number (%)	
*Age*				
10–20	7	2 (1.3)	5 (3.1)	0.50
20–30	48	13 (8.1)	35 (21.8)	
30–40	75	14 (8.8)	61 (38.1)	
40–50	22	8 (5)	14 (8.8)	
50–60	8	2 (1.3)	6 (3.7)	
*Marital status*				
Unmarried	1	1 (100)	0 (0)	0.32
Married	153	37 (24.2)	116 (75.8)	
Widowed	5	1 (20)	4 (80)	
Divorced/separated	1	0 (0)	1 (100)	
*Education*				
Illiterate	55	16 (29.1)	39 (70.9)	0.93
Just literate	7	1 (14.3)	6 (85.7)	
Primary level	27	7 (25.9)	20 (74.1)	
Secondary	41	8 (19.5)	33 (80.5)	
Higher S.	15	3 (20)	12 (80)	
Bachelors	11	3 (27.3)	8 (72.7)	
Master	4	1 (25)	3 (75)	
*Occupation*				
Civil servants	23	7 (30.4)	16 (69.6)	0.36
Farmers	18	7 (38.9)	11 (61.1)	
Business	34	5 (14.7)	29 (85.3)	
Housewife	81	19 (23.5)	62 (76.5)	
Student	4	1 (25)	3 (75)	
*Ethnicity*				
Brahmin	40	10 (25)	30 (75)	0.24
Chhetri	36	7 (19.4)	29 (80.6)	
Janajati	75	17 (22.7)	58 (77.3)	
Dalit	2	1 (50)	1 (50)	
Others^†^	7	4 (57.1)	3 (42.9)	

^†^Include Madhesi, Marwari, Bangali, Jain, and Punjabi/Sikh.

**Table 3:** Distribution of cases according to behavior characteristics.

	Bacterial vaginosis			*P* value
	Number (160)	Positive number (%)	Negative number (%)	
*Smoking*				
Never	140	36 (25.71)	104 (74.29)	0.17
Daily	10	3 (30)	7 (70)	
Occasional	10	0 (0)	10 (100)	
*Alcohol intake*				
Never	129	31 (24)	98 (76)	0.37
Daily	13	5 (38.5)	8 (61.5)	
Occasional	18	3 (16.7)	15 (83.3)	
*Diet*				
Veg	13	2 (15.4)	11 (84.6)	0.43
Nonveg	147	37 (25.2)	110 (74.8)	
*Condom use*				
Never	129	29 (22.5)	100 (77.5)	0.39
Daily	7	3 (42.9)	4 (57.1)	
Sometime	24	7 (29.2)	17 (70.8)	
*Douching*				
Never	38	3 (7.9)	35 (92.1)	0.015^*∗*^
Daily	84	27 (32.1)	57 (67.9)	
Sometime	38	9 (23.7)	29 (76.3)	

^*∗*^ *P* value < 0.05 was considered statistically significant.

**Table 4:** Distribution of BV using different contraceptives.

Contraceptive types	Bacterial vaginosis			*P* value
	Number *N* (160)	Positive number (%)	Negative number (%)	
*Nonanatomical sites*				0.2
OC pills	11	1 (9.1)	10 (90.9)	
Depo-Provera	17	3 (17.6)	14 (82.4)	
Implant	5	1 (20)	4 (80)	
*Anatomical sites*				
Barrier method	30	8 (26.7)	22 (73.3)	
IUCD	10	3 (30)	7 (70)	
Vasectomy (male)	6	3 (50)	3 (50)	
Tubectomy (female)	11	4 (36)	7 (64)	
*Nonusers*	70	16 (22)	54 (78)	

**Table 5:** Sample distribution on the basis of symptoms.

Vaginal symptoms	Bacterial vaginosis			*P* value
	Number *N* (160)	Positive number (%)	Negative number (%)	
*Discharge*				
Normal	61	3 (4.9)	58 (95.1)	0.0001^*∗*^
Thin	83	33 (39.8)	50 (60.2)	
Thick	16	3 (18.8)	13 (81.2)	
*Odor*				
Foul	104	31 (29.9)	73 (70.1)	0.02^*∗*^
Nonfoul	56	8 (14.3)	48 (85.7)	
*Color*				
White	126	34 (27)	92 (73)	0.13
Grey	34	5 (14.7)	29 (85.3)	
*Amount*				
Excess	80	24 (30)	56 (70)	0.09^*∗*^
Scanty	80	15 (18.7)	65 (81.3)	
*Abdominal pain*				
Yes	83	22 (26.6)	61 (73.4)	0.51
No	77	17 (22.1)	60 (77.9)	
*Itching*				
Yes	56	13 (23.2)	43 (76.8)	0.80
No	104	26 (25)	78 (75)	

^*∗*^ *P* value < 0.05 was considered statistically significant.

**Table 6:** Prevalence of microorganisms isolated from vaginal swabs.

Microorganisms	Number (%)
Gram negative:	
*Escherichia coli*	8 (6.3)
*Klebsiella *spp.	4 (3.1)
*Proteus vulgaris*	1 (0.8)
*Proteus mirabilis*	4 (3.1)
*Pseudomonas *spp.	10 (7.8)
*Neisseria gonorrhoeae*	4 (3.1)
*Acinetobacter *spp.	8 (6.3)
*Citrobacter koseri*	1 (0.8)
*Enterobacter *spp.	1 (0.8)
Gram-positive:	
*Streptococcus agalactiae*	7 (5.5)
*Staphylococcus aureus*	7 (5.5)
CoNS	4 (3.1)
*Enterococcus *spp.	5 (3.9)
*Lactobacillus *spp.	35 (27.3)
Others: (yeasts and parasite)	
Yeast:	
*Candida albicans*	3 (2.3)
*Candida *spp.	12 (9.4)
*Trichomonas vaginalis*	14 (10.9)

## Data Availability

All data generated or analyzed during the current study are included in this published article.

## Additional Points

*Limitation of the Study*. Most of the anaerobes associated with BV are fastidious and require selective enriched media, and sometimes they are not easy to identify using traditional biochemical tests. In addition, a number of organisms that seemed associated with BV are uncultivable, for example, species of* Eggerthella*,* Megasphaera*, and BVABs members of Clostridiales. Also, it was demonstrated that, in women of reproductive age group, anaerobic bacteria outnumbered aerobic bacteria; however, the latter appeared to be more abundant with advancing age, onset of sexual activity, and parity of women.
